# Sequestered gravity in gauge mediation

**DOI:** 10.1140/epjc/s10052-016-4192-8

**Published:** 2016-07-01

**Authors:** Ignatios Antoniadis, Karim Benakli, Mariano Quiros

**Affiliations:** 1 Sorbonne Universités, UPMC Univ Paris 06, UMR 7589, LPTHE, 75005 Paris, France; 2 CNRS, UMR 7589, LPTHE, 75005 Paris, France; 3 Albert Einstein Center, Institute for Theoretical Physics, Bern University, Sidlestrasse 5, 3012 Bern, Switzerland; 4 Institut de Fisica d’Altes Energies (IFAE), The Barcelona Institute of Science and Technology (BIST), Campus UAB, 08193 Bellaterra (Barcelona), Spain; 5 Institució Catalana de Recerca i Estudis, Avançats (ICREA), Campus UAB, 08193 Bellaterra (Barcelona), Spain; 6 ICTP South American Institute for Fundamental Research, Instituto de Física Teórica, Universidade Estadual Paulista, São Paulo, Brazil

## Abstract

We present a novel mechanism of supersymmetry breaking embeddable in string theory and simultaneously sharing the main advantages of (sequestered) gravity and gauge mediation. It is driven by a Scherk–Schwarz deformation along a compact extra dimension, transverse to a brane stack supporting the supersymmetric extension of the Standard Model. This fixes the magnitude of the gravitino mass, together with that of the gauginos of a bulk gauge group, at a scale as high as $10^{10}$ GeV. Supersymmetry breaking is mediated to the observable sector dominantly by gauge interactions using massive messengers transforming non-trivially under the bulk and Standard Model gauge groups and leading to a neutralino LSP as dark matter candidate. The Higgsino mass $\mu$ and soft Higgs-bilinear $B_\mu$ term could be generated at the same order of magnitude as the other soft terms by effective supergravity couplings as in the Giudice–Masiero mechanism.

## Introduction

The gravity-mediated supersymmetry breaking scenario with an $\mathcal O(\mathrm{TeV})$ gravitino [1], which can be realized for instance in the minimal supersymmetric extension of the Standard Model (MSSM) has, apart from solving the hierarchy problem, the phenomenological advantages of providing gauge coupling unification at scales $M_\mathrm{GUT}\sim 10^{16}$ GeV and a standard dark matter candidate in the presence of unbroken *R*-parity if the lightest supersymmetric particle (LSP) is a neutralino. Gravity mediation has nevertheless two main drawbacks: (i) Gravitational interactions are not automatically flavor blind and thus they do not guarantee a solution to the supersymmetric flavor problem; (ii) an $\mathcal O(\mathrm{TeV})$ gravitino decays in a lifetime of about $10^6$ s, leading to a huge entropy production after the big bang nucleosynthesis (BBN) and spoiling its predictions unless the reheating temperature after inflation is $\lesssim$ $10^{10}$ GeV, which puts an uncomfortable bound on inflationary scenarios. The latter is known as the *cosmological gravitino problem* [2].

The main motivation for gauge-mediated supersymmetry breaking (see for example [3] and references therein) is that it provides flavor independent soft breaking terms thus avoiding strong experimental constraints on flavor changing neutral currents (FCNC). On the other hand, it also has some problematic drawbacks: (i) One loses the standard dark matter candidate as a WIMP (weakly interacting massive particle) since the LSP is now the gravitino; (ii) The gravitino can be (warm) dark matter only if its mass is $m_{3/2}\lesssim 1$ keV, which requires an upper bound on the messenger mass, *M*, over its number, *N*, as $M/N\lesssim 10^7$ GeV, while for larger gravitino masses the reheating temperature after inflation is strongly constrained; (iii) There is no compelling way to generate a supersymmetric $\mu$-parameter (Higgsino mass) and a $B_\mu$ soft term (Higgs bilinear) of the same order as the other soft terms. Although none of these problems can disqualify gauge mediation as a very appealing mechanism of supersymmetry breaking transmission to the observable sector it would be certainly interesting to find a theory where these problems do not appear.

In this work, we propose a mechanism to solve these problems by appropriately sequestering the supersymmetry breaking in the hidden sector in a string theory context. Due to the sequestering of gravity, even if our gravitino is supermassive gauge-mediated interactions will be dominant over gravity-mediated ones, thus solving the supersymmetric flavor problem. Moreover, since the gravitino mass is $m_{3/2}\gg 1$ TeV our model does not exhibit any gravitino problem and the best candidate for dark matter is the lightest neutralino.

Let us finally mention that the idea of solving some of the specific problems to gauge and/or gravity mediation using a hybrid gauge-gravity mediation mechanism is of course not new. Related studies can be found e.g. in Refs. [4–7]. In all these papers gauge and gravity mediation compete *on the same footing*, the gravitino is in the 100 GeV–1 TeV range, and dark matter can be either the gravitino or the lightest supersymmetric particle. What is new in our approach is that gravitational interactions are sequestered while the gravitino is much heavier, thus decoupling from the spectrum and not generating any kind of cosmological problem.

Of course the idea of sequestering gravitational interactions in gauge-mediated models has already been used in the literature, where two main approaches have been used: (i) There are 4D models with conformal sequestering in the supersymmetry breaking sector [8, 9]. They use the fact that if the supersymmetry breaking sector is strongly coupled, conformal sequestering may lead to flavor violating gravity-mediated operators suppressed by large anomalous dimensions; (ii) in five-dimensional supersymmetric models gravity mediation can be sequestered from gauge mediation provided that the supersymmetry breaking sector and the observable sectors are localized at different branes [10–12]. Gravity mediation effects from the hidden to the observable sector are then exponentially suppressed by the branes separation. In all these models gravity is sequestered from the gauge interactions of gauge mediation, a heavy enough gravitino with a mass range 100 GeV–100 TeV is obtained and the LSP, and Dark Matter candidate, is the lightest neutralino. However, our approach is different and simpler in many aspects: supersymmetry breaking is a global effect in the bulk, via Scherk–Schwarz twisted boundary conditions, leading to finite radiative corrections, and the gravitino, which automatically decouple from the low energy spectrum, is superheavy $\sim$ $10^{10}$ GeV and safe from all kind of cosmological problems. Moreover, LSP is the lightest neutralino, by which it is also a good candidate to Dark Matter, and our results on the string scale are consistent with the MSSM gauge coupling unification conditions.

Our basic setup is the following. Motivated by type I string theory constructions, we consider the MSSM localized in three (spatial) dimensions, on a collection of *D*-brane stacks, which we call in short a 3-brane, transverse to a “large” extra dimension on a semi-circle (orbifold) of radius *R*, along which there is a bulk “hidden” gauge group $G_\mathrm{H}$ associated to another (higher-dimensional) brane. We assume that $G_\mathrm{H}$ has a non-chiral spectrum. There are in general matter fields, described by excitations of open strings stretched between the Standard Model (SM) brane and the brane extended in the bulk, and thus localized in their three-dimensional (spatial) intersection. They transform in the corresponding bi-fundamental representations and, since they are non-chiral, they can acquire a mass *M* by appropriate brane displacements (or equivalently Wilson lines) that we consider as a parameter of the model. They will play the role of messengers to transmit supersymmetry breaking to the observable sector.

Supersymmetry breaking is induced by a Scherk–Schwarz (SS) deformation along the extra dimension generating a Majorana mass for fermions in the bulk, namely the gravitino and the gauginos of $G_\mathrm{H}$, proportional to the compactification scale 1 / *R*, but leaving the SM brane supersymmetric [13]. The breaking is mediated to the observable SM sector by both gravitational [13, 14] and gauge interactions [15] (via the bi-fundamental messenger fields), whose relative strength is controlled by the compactification scale and messenger mass. Fixing for definiteness the MSSM soft terms at the TeV scale and requiring gravitational contributions to the squared scalar masses to be suppressed, with respect to the gauge-mediated ones, between two and four orders of magnitude,^1^ one finds that the compactification scale 1 / *R* should be less than about $1/R \sim 10^{10}$ GeV, corresponding to a string scale in the unification region $M_\mathrm{GUT}\sim 10^{16}$ GeV, inferred by extrapolating the low energy SM gauge couplings with supersymmetry.

The resulting MSSM soft terms (sfermion and gaugino masses) have then the usual pattern of gauge mediation, with in particular scalar masses that are essentially flavor blind, guaranteeing the absence of dangerous flavor changing neutral current interactions. Note thought that in contrast to the standard gauge mediation scenario, the gravitino mass is heavy, of order the compactification scale, evading the gravitino overproduction problem and having as LSP the lightest neutralino, like in models of gravity mediation, in the right ballpark needed for describing the missing dark matter of the Universe required by astrophysical and cosmological observations. On the other hand, a globally supersymmetric Higssino mass $\mu$ term and its associated Higgs-bilinear soft term $B_\mu$ can be generated in a similar way as in the Giudice–Masiero mechanism [16], by effective supergravity *D*-term interactions involving a non-holomorphic function depending on the radius modulus field *T* whose *F*-auxiliary component acquires a non-vanishing expectation value, as dictated by the SS deformation. Under a reasonable assumption on the asymptotic dependence, the induced $\mu$ and $B_\mu$ parameters are of the same order with the rest of the MSSM soft supersymmetry breaking terms.

The outline of the paper is the following. In Sect. 2 we compute the contribution of gravity mediation to scalar and gaugino masses and we define the upper bound of the compactification scale in order to suppress the former contribution from the total. In Sect. 3 we compute the corresponding contribution of gauge mediation and determine the region of messenger mass that leads to a viable phenomenological spectrum. In Sect. 4 we discuss the generation of $\mu$ and $B_\mu$ terms, while Sect. 5 contains our conclusions. Finally, in Appendix A, we present the details of the computation of the induced *F*-auxiliary expectation value in the messenger sector off-shell, needed for the evaluation of the gauge-mediated contributions in the main text.

## Gravity mediation for SS supersymmetry breaking

Our starting setup is a higher-dimensional space where the MSSM is localized on a D3-brane that is perpendicular to a large compact coordinate (of radius *R*). Supersymmetry is assumed to be broken by a Scherk and Schwarz [17, 18] mechanism giving to the gaugino $\lambda _\mathrm{H}$ and gravitino Kaluza–Klein (KK) modes a common mass:

2.1 $$\begin{aligned} M_{n}(\omega )=m_{3/2}+\frac{n}{R},\quad m_{3/2}=\frac{\omega }{R} \end{aligned}$$

where $m_{3/2}$ is the mass of the gravitino zero mode and $\omega$ a real parameter $0<\omega <\frac{1}{2}$.

We are interested here in evaluating the size of the MSSM supersymmetry breaking soft terms mediated by gravitational (grav) effects $(m_0^\mathrm{grav },M_{1/2}^\mathrm{grav})$, which we shall compare in the next section to those from gauge interactions. Supersymmetry breaking is transmitted from the bulk to the brane by one-loop gravitational interactions giving a (squared) mass to scalars proportional to [14, 19]

2.2 $$\begin{aligned} \left( m_0^\mathrm{grav}\right) ^2=\frac{1}{M_\mathrm{P}^2}\sum _n\int \frac{\mathrm{d}^4k}{(2\pi )^4}k^2\left[ \frac{1}{k^2+M_n^2(0)}-\frac{1}{k^2+M_n^2(\omega )}\right] , \end{aligned}$$

where $M_\mathrm{P}=2.4\times 10^{18}$ GeV is the reduced Planck mass, and a Majorana mass to gauginos proportional to

2.3 $$\begin{aligned} M_{1/2}^\mathrm{grav}=\frac{1}{M_\mathrm{P}^2}\sum _n\int \frac{\mathrm{d}^4k}{(2\pi )^4}\left[ \frac{M_n(\omega )}{k^2+M_n^2(\omega )}-\frac{M_n(0)}{k^2+M_n^2(0)}\right] . \end{aligned}$$

In particular the gravitational correction to the squared mass of localized matter scalars $\varphi$ is given by

2.4 $$\begin{aligned} m_{\varphi \bar{\varphi }}^2=G^{-1}_{\varphi \bar{\varphi }}\left( G^{i\bar{j}}R_{i\bar{j} \varphi \bar{\varphi }}-G_{\varphi \bar{\varphi }} \right) \left( m_0^\mathrm{grav}\right) ^2 \end{aligned}$$

where $G_{i\bar{j}}$ and $G_{\varphi \bar{\varphi }}$ are the moduli and matter metrics, respectively, while $R_{i\bar{j} \varphi \bar{\varphi }}$ is the moduli–matter Riemann tensor. The factor $G^{-1}_{\varphi \bar{\varphi }}$ comes from the wave function renormalization and the two terms in the bracket in Eq. (2.4) come from the moduli and graviton supermultiplets, respectively. Then the total contribution to $m_{\varphi \bar{\varphi }}^2$ has completely different pattern in flavor space depending on the behavior of different moduli. We will then consider two different general cases depending on moduli masses $m_{i\bar{i}}^2$ provided by the moduli stabilization mechanism.

(i) Heavy moduli: if $m_{i\bar{i}}^2>1/R^2$, moduli decouple from the low energy effective theory and the first term in Eq. (2.4) does not contribute. In this case, even for anarchic matter metric, after diagonalization of the matter kinetic terms the gravitational corrections are flavor diagonal.
(ii) Light moduli: if $m_{i\bar{i}}^2<1/R^2$, moduli do not decouple from the low energy effective theory and the contribution of the Riemann tensor in the first term of Eq. (2.4) can create different moduli dependent contribution to different scalar fields triggering dangerous flavor changing neutral currents.

The gravitational squared mass of scalars can then be expressed as

2.5 $$\begin{aligned} \left( m_0^\mathrm{grav}\right) ^2=\frac{1}{R^2}\frac{1}{(M_\mathrm{PR})^2}\frac{1}{(4\pi )^2} f_0(\omega ) \end{aligned}$$

where

2.6 $$\begin{aligned} f_0(\omega )=\frac{3}{2 \pi ^4}\left[ 2\zeta (5)-Li_5(e^{2i\pi \omega })-Li_5(e^{-2i\pi \omega }) \right] \end{aligned}$$

and the gravitational Majorana gaugino mass as

2.7 $$\begin{aligned} M_{1/2}^\mathrm{grav}=\frac{1}{R}\frac{1}{(M_\mathrm{PR})^2}\frac{1}{(4\pi )^2} f_{1/2}(\omega ) \end{aligned}$$

where

2.8 $$\begin{aligned} f_{1/2}(\omega )=\frac{3i}{8\pi ^3}\left[ Li_4(e^{-2i\pi \omega })-Li_4(e^{2i\pi \omega }) \right] . \end{aligned}$$

**Fig. 1:**
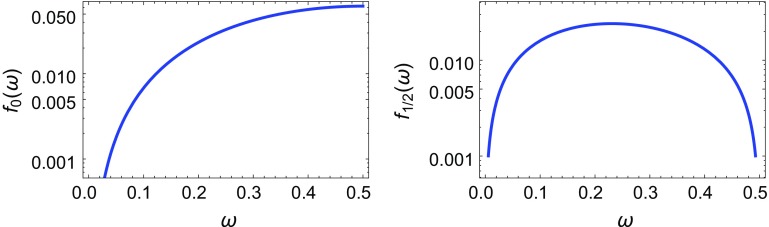
*Left panel* Plot of $f_0(\omega )$. *Right panel* Plot of $f_{1/2}(\omega )$

Note that $f_{1/2}(\omega )$ vanishes for $\omega = \frac{1}{2}$ because of a preserved *R*-symmetry [14, 19]. As we focus on models with Majorana masses for gauginos, we need therefore to take $\omega \in (0, 1/2 )$. For $\omega \sim 1/2$ there are two quasi-degenerate Majorana gravitinos, one of which couples to the MSSM (the even parity one) at the 3-brane location. The functions $f_0(\omega )$ and $f_{1/2}(\omega )$ are plotted in Fig. 1, left and right panels, respectively, where we can see that their values are typically $\mathcal O(10^{-2})$.

**Fig. 2:**
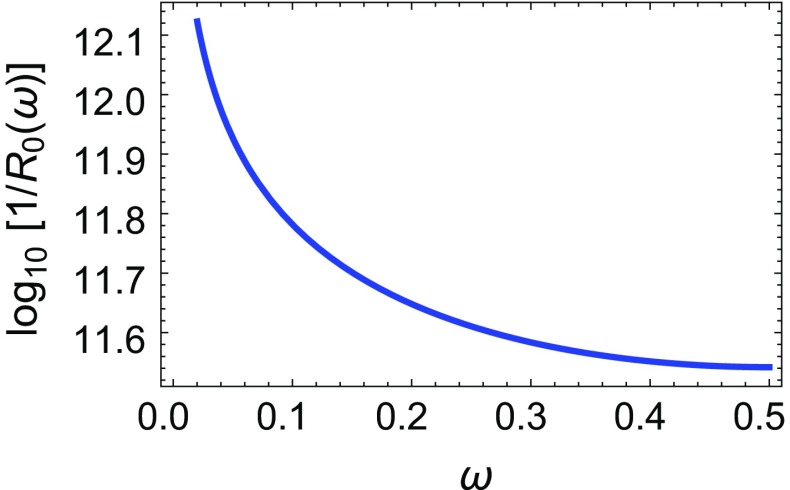
Plot of $\log _{10}[1/R_0(\omega )/\mathrm{GeV}]$ by fixing $|m_0^\mathrm{grav}|=1$ TeV

The gravitational contributions to the scalar squared masses are (in the absence of other moduli mediating supersymmetry breaking) negative and flavor diagonal as they are mediated by diagrams with gravitinos and gravitons exchanged in the loops [14]. One can always fix the radius to a reference value $R_0$ by imposing the condition that $|m_0^\mathrm{grav}|\simeq 1$ TeV. The result $1/R_0(\omega )$ is plotted in Fig. 2 where we can see that $(1/R_0)\sim 10^{12}$ GeV (almost) independently of the value of $\omega$. For the corresponding values of the gravitino mass [$m_{3/2}\equiv \omega /R_0(\omega )$] the Majorana gaugino masses are small enough to be neglected. Even in the presence of flavor non-diagonal matter Kahler metrics, for larger radii the gravitational contributions to scalar masses can become negligible, so if there exists another (gauge) mediation mechanism of supersymmetry breaking to the observable sector which generates flavor conserving leading contributions to squared scalar masses $(m_0^\mathrm{gauge})^2$, as will be described in Sect. 3, we will require that flavor non-conserving gravity-mediated contributions $(\Delta m_0^\mathrm{grav})^2$ do not account for more than one per mille of soft squared masses at the mediation scale.

In fact bounds on flavor changing processes should provide bounds on the parameter

2.9 $$\begin{aligned} \delta \equiv \frac{(\Delta m_0^\mathrm{grav})^2}{(m_0^\mathrm{gauge})^2}\ . \end{aligned}$$

Under the assumption of an anarchic gravitationally induced Kahler metric, the parameter $\delta$ is constrained by the strongest flavor constraints, which correspond to the *CP*-violating observable $\epsilon _K$ generated by the imaginary part of the $\Delta F=2$ effective dimension-six operator $(1/\Lambda _F^2)(\bar{s}_R d_L)(\bar{s}_L d_R)$ for which $\Lambda _F\gtrsim 4\times 10^5$ TeV [20–22]. This provides, depending on the masses of the supersymmetric spectrum and the scale of supersymmetry breaking, different bounds on the parameter $\delta$.

(i) Heavy moduli: For the previously introduced case where moduli are decoupled from the low energy effective theory, gravitational contributions to scalar masses are only from the graviton multiplet and thus flavor diagonal at one-loop even in the presence of anarchic matter metric. In this case, and considering potential higher-loop suppressed contributions which could be flavor non-diagonal, a conservative bound on $\delta$, as $\delta \lesssim 10^{-2}$, will be imposed.
(ii) Light moduli: For the case where the presence of moduli could induce FCNC a stronger constraint should be imposed. For that purpose we will use the conservative bound $\delta \lesssim 10^{-4}$, which we will consider later on in this paper.^2^

There is also a contribution that originates from anomaly mediation (AM) and that provides scalar and gaugino masses as [23, 24]

2.10 $$\begin{aligned} m_0^{AM}\sim M_{1/2}^{AM}\sim \frac{\lambda ^2}{16\pi ^2}F_\phi \end{aligned}$$

where $\lambda$ indicates the different four-dimensional gauge and Yukawa couplings and $F_\phi$ is the *F*-component of the chiral compensator $\phi =1+\theta ^2 F_\phi$. In the case of a Scherk–Schwarz breaking it turns out that, as in no-scale models, $F_\phi \ll m_{3/2}$. In fact one can prove that at the tree level $F_\phi =0$ [25] while at one-loop a gravitational Casimir energy $V\sim (1/16\pi ^2) 1/R^4$ is generated which yields a non-zero value of $F_\phi$ as $F_\phi \sim (1/16\pi ^2) m_{3/2}^3/M_\mathrm{P}^2$ [26, 27]. Therefore its contribution to scalar masses from (2.10) is negligible, as compared to the gravitational contribution, Eq. (2.2), while its contribution to gaugino masses is of the same order of magnitude as the gravitational ones, Eq. (2.3).

## Gauge mediation for SS supersymmetry breaking

We now turn to compute the supersymmetry breaking effects induced by the bulk-brane gauge interactions. We will denote as $G_\mathrm{H}$ the gauge symmetry group living in the bulk and by $\alpha _\mathrm{H}$ the associated four-dimensional coupling.^3^ We consider a number of messengers $(\phi _I,\bar{\phi }_I)$ living in the intersection between the MSSM 3-brane and the brane in the bulk which contains the SS direction. The messengers transform under the representation $(\mathcal R^{G_\mathrm{H}}_{\phi _I},\, \overline{\mathcal R}^{G_\mathrm{H}}_{\phi _I})$ of the hidden gauge group $G_\mathrm{H}$ and under the representation $(\mathcal R^{G_\mathrm{SM}}_{\phi _I},\, \overline{\mathcal R}^{G_\mathrm{SM}}_{\phi _I})$ of the Standard Model group $G_\mathrm{SM}$.

### The messenger sector

By the SS supersymmetry breaking the gauginos of the hidden gauge group $\lambda _\mathrm{H}$ acquire, as the gravitinos, soft Majorana masses:

3.1 $$\begin{aligned} M_{n}(\omega )=M_{1/2}+\frac{n}{R},\quad M_{1/2}=m_{3/2}=\frac{\omega }{R}. \end{aligned}$$

We assume, as in ordinary gauge mediation, that the messengers have a supersymmetric mass as

3.2 $$\begin{aligned} W=\phi _I\,M_{IJ}\,\bar{\phi }_J \end{aligned}$$

where, without loss of generality, the mass matrix is diagonal $M_{IJ}=M_I\delta _{IJ}$, and we are assuming that $M_I\simeq M$ ($\forall I$). This diagonal mass matrix will induce a diagonal supersymmetry breaking parameter, denoted by $F_I=\lambda _I F$, through the one-loop radiative corrections induced by the bulk gaugino $\lambda _\mathrm{H}$ and Dirac fermion $(\widetilde{\phi }_I,\widetilde{\bar{\phi }}_\mathrm{I})$, that is, proportional to [28].^4^ We have

3.3 $$\begin{aligned} F= & {} \frac{\alpha _\mathrm{H}}{\pi } \, \int _0^\infty \mathrm{d}p^2\, p^2\sum _n\left[ \frac{M_n(\omega )}{p^2+M_n^2(\omega )} -\frac{M_n(0)}{p^2+M_n^2(0)} \right] \nonumber \\&\times \frac{M}{p^2+M^2} \end{aligned}$$

where $\lambda _\mathrm{I}=C^{G_\mathrm{H}}_{\phi _\mathrm{I}}$ is the quadratic Casimir operator of the representation $\mathcal R^{G_\mathrm{H}}_{\phi _\mathrm{I}}$ of $G_\mathrm{H}$. The integral has different behaviors for $\mathrm{RM}<1$ and $\mathrm{RM}>1$. In fact it can be written as

3.4 $$\begin{aligned}&F=\frac{\alpha _\mathrm{H}}{4\pi } \frac{M}{R}\left\{ \begin{array}{lll} g_0(\omega ), &{} \mathrm{for}\quad \mathrm{RM}\ll 1:&{} g_0(\omega )=2i\left[ Li_2(r)-Li_2(1/r) \right] /\pi \\ &{}&{} \\ 1/\left( MR \right) ^2\, g_\infty (\omega ), &{} \mathrm{for}\quad \mathrm{RM}\gg 1:&{} g_\infty (\omega )=3i\left[ Li_4(r)-Li_4(1/r) \right] /\pi ^3 \end{array} \right. \end{aligned}$$

**Fig. 3:**
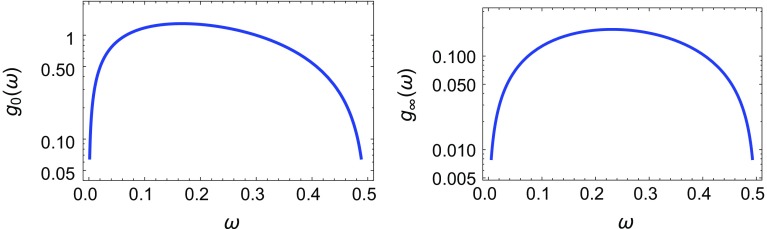
*Left panel* Plot of $g_0(\omega )$. *Right panel* Plot of $g_{\infty }(\omega )$

where $r=e^{-2i\pi \omega }$. The functions $g_0(\omega )$ and $g_\infty (\omega )$ are plotted in Fig. 3 where we see that they satisfy the relation $g_\infty (\omega )\sim 0.1 g_0(\omega )$. A quick glance at Eq. (3.4) shows that the parameter *F* / *M* is larger for $M\lesssim 1/R$ than for $M\gtrsim 1/R$ so that in the following we will only consider the former case of $g_0(\omega )$.

### The observable sector

Supersymmetry breaking is then transmitted through the usual gauge mediation mechanism [3] to squark, slepton and gaugino masses of the MSSM. In order to not spoil the MSSM gauge coupling unification we can assume that the messenger sector consists in complete *SU*(5) representations. For instance we can assume $n_5$ multiplets in the $5+\bar{5}$, $[(D,L)+(\bar{D},\bar{L})]$, and $n_{10}$ multiplets in the $10+\overline{10}$, $[(Q,U,E)+(\bar{Q},\bar{U},\bar{E})]$. The mass generated by gauge mediation for gauginos and scalars can then be written as

3.5 $$\begin{aligned} M_3= & {} \frac{\alpha _3}{4\pi }\left[ n_5\Lambda _D+n_{10}(2\Lambda _Q+\Lambda _U ) \right] ,\nonumber \\ M_2= & {} \frac{\alpha _2}{4\pi }\left[ n_5\Lambda _L+3n_{10}\Lambda _Q \right] ,\nonumber \\ M_1= & {} \frac{\alpha _1}{4\pi } \frac{6}{5}\left[ n_5\left( \frac{1}{3}\Lambda _D +\frac{1}{2}\Lambda _L \right) \right. \nonumber \\&\left. +n_{10}\left( \frac{1}{6}\Lambda _Q+\frac{4}{3}\Lambda _U+\Lambda _E \right) \right] , \end{aligned}$$

where $$\Lambda _\mathrm{I}= C_\mathrm{I}^{G_\mathrm{H}} F/M$$ ($$I=D,L,Q,U,E$$), and

3.6 $$\begin{aligned} m_{\tilde{f}}^2=2\left[ C_3^{\tilde{f}}\left( \frac{\alpha _3}{4\pi }\right) ^2 \Lambda _3^2+C_2^{\tilde{f}}\left( \frac{\alpha _2}{4\pi }\right) ^2 \Lambda _2^2+C_1^{\tilde{f}}\left( \frac{\alpha _1}{4\pi }\right) ^2 \Lambda _1^2 \right] \end{aligned}$$

where $C_i^{\tilde{f}}$ is the quadratic Casimir operator of the $\tilde{f}$ representation with the normalization $C_1^f=\frac{3}{5}Y_f^2$, and where all couplings are at the scale *M*. Similarly the scales $\Lambda _i$ are defined as

3.7 $$\begin{aligned} \Lambda _3^2&=n_5\Lambda _D^2+n_{10}(2\Lambda _Q^2+\Lambda _U^2 ),\nonumber \\ \Lambda _2^2&= n_5\Lambda _L^2+3n_{10}\Lambda _Q^2,\nonumber \\ \Lambda _1^2&=\frac{6}{5}\left[ n_5\left( \frac{1}{3}\Lambda _D^2 +\frac{1}{2}\Lambda _L^2 \right) +n_{10}\left( \frac{1}{6}\Lambda _Q^2+\frac{4}{3}\Lambda _U^2+\Lambda _E^2 \right) \right] . \end{aligned}$$

To simplify the analysis we will assume that the structure of *SU*(5) multiplets is not spoiled by $$G_\mathrm{H}$$ so that $$\Lambda _\mathrm{I}\equiv \Lambda _5=C_5^{G_\mathrm{H}}\, F/M$$ ($$ I=D,L$$) and $$\Lambda _\mathrm{I}\equiv \Lambda _{10}=C_{10}^{G_\mathrm{H}}\, F/M$$ ($$ I=Q,U,E$$)^5^ in which case the previous equations yield

3.8 $$\begin{aligned} M_i&=\frac{\alpha _i}{4\pi }\left[ n_5\Lambda _5+3 n_{10}\Lambda _{10} \right] ,\nonumber \\ \Lambda _i^2&=n_5\Lambda _5^2+3 n_{10}\Lambda _{10}^2. \end{aligned}$$

As we want the LSP, and thus the dark matter component of the universe, to be a well tempered Bino/Higgsino admixture ($\tilde{B}/\tilde{H}$) we need sfermions to be heavier than $M_1$ at the low scale, a condition which prevents a large number of messengers (as we will next see). So from Eq. (3.8) it is obvious that the case where only the number $n_5$ of $5+\bar{5}$ is charged under $G_\mathrm{H}$, i.e. $C_5^{G_\mathrm{H}}\ne 0$, while the messengers in the $10+\overline{10}$ are neutral under $G_\mathrm{H}$ and thus $C_{10}^{G_\mathrm{H}}=0$ (i.e. $\Lambda _{10}=0$), is preferred as $n_{10}$ has multiplicity three in (3.8). In this case we obtain the usual expressions of minimal gauge mediation

3.9 $$\begin{aligned} M_i&=\frac{\alpha _i}{4\pi }\Lambda _G,\quad \Lambda _G=n_5\Lambda _5,\nonumber \\ \Lambda _i^2&=\Lambda _S^2,\quad \Lambda _S^2=n_5\Lambda ^2_5, \end{aligned}$$

and we should keep $n_5$ as small as possible. Moreover, to keep the multiplicity of the representations $\mathcal R^{G_\mathrm{H}}_5$ to the lowest possible values we will assume that $G_\mathrm{H}=U(1)_\mathrm{H}$.

**Fig. 4:**
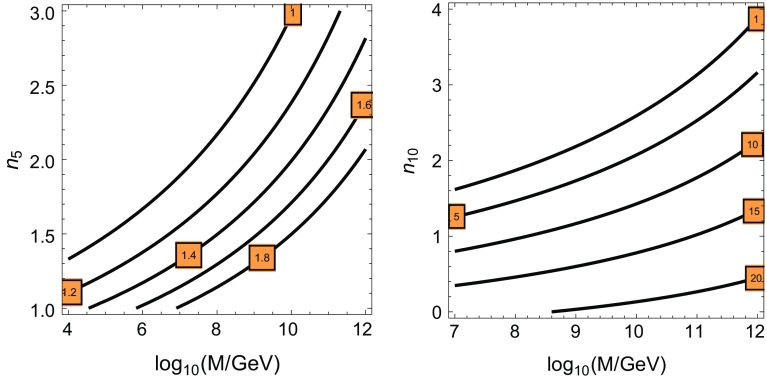
*Left panel* Contour lines of $m^2_{\tilde{\ell }_R}(\mu _0)/M_1^2(\mu _0)$ in the plane $(\log _{10}M/GeV,n_5)$. *Right panel* Contour lines of $1/\alpha _\mathrm{GUT}$ in the plane $(\log _{10}M/GeV,n_{10})$ for $n_5=2$, where $n_{10}$ is the number of messengers in the $10+\overline{10}$ of *SU*(5) uncharged under $G_\mathrm{H}$

In this framework, as the masses are generated at the scale *M* and run to the scale $\mu _0\sim \mathcal O(\text {TeV})$ by the renormalization group equations, the lightest gaugino is the *U*(1) Bino and the lightest scalar is the right-handed slepton $\tilde{\ell }_R$.^6^ As the lightest supersymmetric particle (LSP) is stable^7^ we need the Bino ($\tilde{B}$ with mass $\sim M_1(\mu _0)$) to be lighter than $\tilde{\ell }_R$ as we already mentioned. In this case we would also need the neutral Higgsino $\tilde{H}$ Dirac mass $\mu$ (see next section) to be $\mu \sim M_1$ to avoid the over-closure of the Universe and predict the thermal dark matter density measured by WMAP. This is the so-called well tempered $\tilde{B}/\tilde{H}$ scenario [29]. The conditions for the Bino to be lighter than $\tilde{\ell }_R$ are shown in the left panel of Fig. 4 where we plot contour lines of the ratio $m^2_{\tilde{\ell }_R}(\mu _0)/M_1^2(\mu _0)$ in the plane ($\log _{10}M/GeV,n_5)$.

We can see that for $M\lesssim 10^8$ GeV there is the bound $n_5\le 2$. In particular for $n_5=1$ the value of *M* is not constrained by the LSP requirement, and for $n_5=2$ we have $M\gtrsim 10^7$ GeV. Finally for $n_5=3$ we have $M\gtrsim 10^{10}$ GeV, which essentially saturates the bound obtained in Appendix A; see Eq. (3.17).^8^ All these predictions are independent on the value of $n_{10}$ as we are assuming $C_{10}^{G_\mathrm{H}}=0$ and thus the $10+\overline{10}$ consists in a supersymmetric sector. As shown in the right panel of Fig. 4, where we show contour lines of constant $1/\alpha _\mathrm{GUT}$ in the plane $(\log _{10}M/\mathrm{GeV},n_{10})$ for $n_5=2$, their presence will modify the value of the unification gauge coupling $\alpha _\mathrm{GUT}$ and the model predictions.

There is another way of avoiding the constrains provided by the left plot of Fig. 4. It consists in assuming that the LSP is mainly Higgsino-like with a (heavy) mass $\mu (\mu _0)\sim 1$ TeV [29] so that it would be possible to have $\mu (\mu _0) \lesssim m_{\tilde{\ell }_R}(\mu _0)\lesssim M_1(\mu _0)$. However, in this case $M_1\gtrsim 1$ TeV which would imply, in the minimal gauge mediation scenario we are assuming in this paper, a very heavy gluino $M_3\gtrsim 5$ TeV, and thus a quite heavy supersymmetric spectrum.

### Numerical results

To explicitly compute the value of $\Lambda _S$ we will use the fact that $\alpha _\mathrm{H}$ is the four-dimensional gauge coupling of $G_\mathrm{H}$, so its value is given by [30]

3.10 $$\begin{aligned} \alpha _\mathrm{H}=2\alpha _\mathrm{GUT}/(\mathrm{RM}_s) \end{aligned}$$

where $\alpha _\mathrm{GUT}$ is the SM coupling at the unification scale, and we defined $M_s$ to be the fundamental (string) scale. The value of $\alpha _\mathrm{H}$ can be written in terms of the reduced 4D Planck scale $M_\mathrm{P}=2.4\times 10^{18}$ GeV, using the relation [30]

3.11 $$\begin{aligned} M_\mathrm{P}^2=\frac{M_s^3R}{8\pi \alpha _\mathrm{GUT}^2} (M_sR_T)^{d_T} \end{aligned}$$

where we have included a possible additional number $d_T$ of extra dimensions (where the group $G_\mathrm{H}$ does not propagate) with radii $R_T$ slightly larger than the string length $\ell _s=1/M_s$. This situation can be pictured for instance in type I strings where the SM would correspond to states localized on a *D*3-brane and the large SS dimension inside a *D*7-brane, in which case $d_T=2$.

We can then write

3.12 $$\begin{aligned} \Lambda _S=K_\mathrm{H} g_0(\omega ) \left( 1/R\right) ^{5/3}\left( 1/M_\mathrm{P}\right) ^{2/3} (M_sR_T)^{d_T/3} \end{aligned}$$

where the pre-factor $K_\mathrm{H}$ given by

3.13 $$\begin{aligned} K_\mathrm{H}=\sqrt{n_5}\left( \frac{\alpha _\mathrm{GUT}}{64\,\pi ^4} \right) ^{1/3} C^{G_\mathrm{H}}_\phi \simeq 10^{-1.1}\, \sqrt{\frac{n_5}{2}}\, \alpha _\mathrm{GUT}^{1/3} C^{G_\mathrm{H}}_\phi \end{aligned}$$

contains all the model dependence on the hidden sector.

**Fig. 5:**
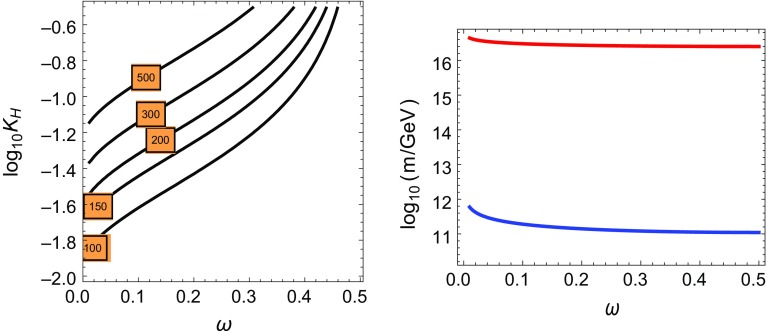
*Left panel* Contour lines of constant $\Lambda _S$ (in TeV) in the plane $(\omega ,K_\mathrm{H})$ for the case of heavy moduli. We have fixed $\delta =10^{-2}$ and no largish extra dimensions, $R_T M_s=1$. *Right panel* Plot of $\log _{10}[1/R(\omega )/\mathrm{GeV}]$ (*lower line*) and $\log _{10}[M_s/\mathrm{GeV}]$ (*upper line*) as functions of $\omega$. For the *upper line* we have considered the case $\alpha _\mathrm{GUT}=\mathcal O(1)$.

**Fig. 6:**
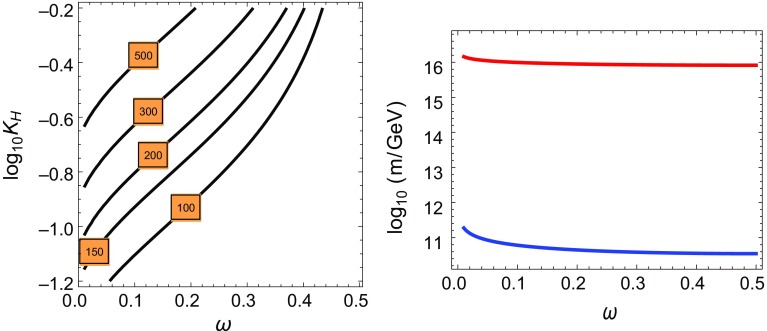
*Left panel* Contour lines of constant $\Lambda _S$ (in TeV) in the plane $(\omega ,K_\mathrm{H})$ for the case of light moduli. We have fixed $\delta =10^{-4}$ and $d_T=2$, $R_TM_s=3$. *Right panel* Plot of $\log _{10}[1/R(\omega )/\mathrm{GeV}]$ (*lower line*) and $\log _{10}[M_s/\mathrm{GeV}]$ (*upper line*) as functions of $\omega$. For the *upper line* we have considered the case $\alpha _\mathrm{GUT}=\mathcal O(1)$

We will fix the radius as $1/R(\omega )\equiv \delta ^{1/4} /R_0(\omega )$, where $R_0(\omega )$ is the value of the radius fixed by the plot in Fig. 2 and $\delta$ is the suppression coefficient which should make the gravitational contribution to the squared scalar masses negligible, as compared to those obtained from gauge mediation. In fact, as explained above, we will require that gravity mediation contributions do not spoil the flavor blindness of the gauge mediation mechanism.

For our numerical estimates we have fixed the value of $\delta$ depending on the two cases concerning moduli masses which we have defined in the previous sections.

(i) Heavy moduli: For decoupled moduli we have considered all extra dimensions (apart from the SS breaking one) small, with values equal to $\ell _s$ (i.e. $R_T M_s=1$) and fixed the parameter $\delta$ to the value $\delta =10^{-2}$ in Fig. 5. In this case the relationship for the scales $M_\mathrm{P}$ and $\Lambda _S$ is given by
  3.14 $$\begin{aligned} M_\mathrm{P}^2=&\frac{M_s^3R}{8\pi \alpha _\mathrm{GUT}^2} \nonumber \\ \Lambda _S=&K_\mathrm{H} g_0(\omega ) \left( 1/R\right) ^{5/3}\left( 1/M_\mathrm{P}\right) ^{2/3} \end{aligned}$$
(ii) Light moduli: For non-decoupled moduli we have assumed $d_T=2$ largish extra dimensions with radii larger than $\ell _s$ by a factor $R_T M_s=\mathcal O(\text {few})$ and the value $\delta =10^{-4}$ in Fig. 6. In this case the relationship for the scales $M_\mathrm{P}$ and $\Lambda _S$ is given by
  3.15 $$\begin{aligned} M_\mathrm{P}^2=&\frac{M_s^3R}{8\pi \alpha _\mathrm{GUT}^2} (M_sR_T)^{2}\nonumber \\ \Lambda _S=&K_\mathrm{H} g_0(\omega ) \left( 1/R\right) ^{5/3}\left( 1/M_\mathrm{P}\right) ^{2/3} (M_sR_T)^{2/3}. \end{aligned}$$

The contour plot for fixed values of $\Lambda _S$ (in TeV) is plotted for $\delta =10^{-2}$ (and $d_T=0$) in the left panel of Fig. 5, and for $\delta =10^{-4}$ in the left panel of Fig. 6, for $d_T=2$ and $R_T M_s=3$, in the plane $(\omega ,K_\mathrm{H})$. The preferred value of $\Lambda _S$ can be obtained from the lower experimental limit on the gluino mass $M_3\gtrsim 1.5$ TeV, which translates into the bound $M_1\gtrsim 250$ GeV for our minimal gauge mediation.^9^ Then from Eq. (3.9) we can extract the value $\Lambda _S=4\pi M_3/(\sqrt{n_5}\alpha _3(M_3))\gtrsim 230\text { TeV}/\sqrt{n_5}$. This value imposes constraints on the hidden sector parameter $K_\mathrm{H}$, which should be as large as possible. First of all we see that the value of $K_\mathrm{H}$ depends on the value of the unification coupling constant and the number of messengers which are non-singlets under the group $G_\mathrm{H}$. The gauge coupling unification value for the MSSM is $\alpha _\mathrm{GUT}^\mathrm{MSSM}\simeq 1/24$ for a unification scale $M_\mathrm{GUT}\simeq 2\times 10^{16}$ GeV. The possible presence of $n_{10}$ states in complete representations of *SU*(5) increases the value of $\alpha _\mathrm{GUT}$ leaving (at one-loop) the value of the unification scale unmodified, as it was shown in the right panel of Fig. 4.

**Fig. 7:**
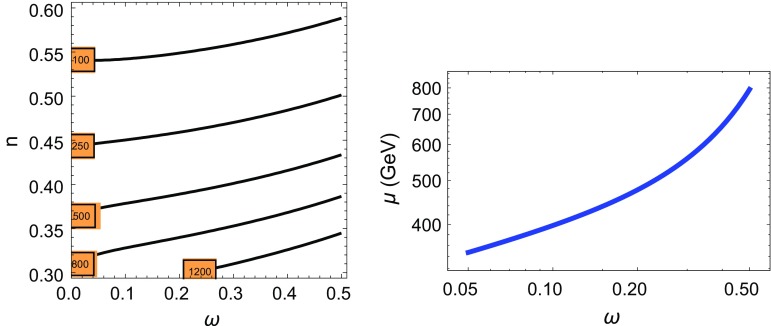
*Left panel* Contour lines of $|\mu |$ in GeV for $a=b=1$ in the plane $(\omega ,n)$. *Right panel* Plot of $|\mu (\omega )|$ for the case $f(T)=\log (T)$ for $a\simeq b\simeq 1/32\pi ^2$

After fixing $\delta$ we are then left with the parameters:

3.16 $$\begin{aligned} M, \quad n_5, \quad n_{10}, \quad \text {and} \quad C_5^{G_\mathrm{H}}. \end{aligned}$$

In the simplest framework of minimal gauge mediation considered here, and as from the previous considerations, we are left to consider the cases of $n_5=1,2$. Moreover, the messenger mass *M* is constrained to be of order

3.17 $$\begin{aligned} M\lesssim \left( 0.1/R\right) \sim 10^{10} {\text { GeV}} \end{aligned}$$

from the validity of our approximation for *F*, as explained in Appendix A, combined with a conservative value of $\left( 1/R_0\right)$ extracted from Fig. 2. Notice that the requirement $F/M^2<1$ (a condition to avoid tachyons in the messenger sector in gauge mediation) is always satisfied because of the smallness of $\alpha _\mathrm{H}$. Clearly, $C^{G_\mathrm{H}}_5$ has a strong impact on the value of $K_\mathrm{H}$ and the spectrum of the model. This is given by the squared of the charge of the messengers under $U(1)_\mathrm{H}$, $Q_\mathrm{H}^2$. Typical values could be for example $C^{G_\mathrm{H}}_5 =1$ ($Q_5=\pm 1$), in which case the model is more contrived as we shall discuss shortly, or $C^{G_\mathrm{H}}_5=4$ ($Q_5=\pm 2$), which gives more room for the other parameters. Examples of models with extra *U*(1)’s and their charges can be found in the literature; see e.g. Ref. [32].

For $$C^{G_\mathrm{H}}_5=1$$, the necessary values of $$K_\mathrm{H}$$ require larger values of $$\alpha _\mathrm{GUT}$$. This could be achieved by increasing $$n_5$$ and/or $$n_{10}$$ and lowering *M*. However, keeping the Bino as the LSP requires instead lower $$n_5$$, and higher *M*, values, which creates a tension. Hence, we choose to keep *M* in the range $$10^{8}$$–$$ 10^{10}$$ GeV and introduce a number of neutral messengers, $$n_{10} > 0$$. For example, for $$\delta =10^{-2}$$ valid sets $$[M , n_5, n_{10}, \alpha _\mathrm{GUT}]$$ leading to phenomenologically viable spectra are e.g. $$[10^8 \text { GeV}, 1, 1, 1/10]$$ or $$[10^7 \text { GeV}, 2, 2, 1]$$. A larger range on the parameters would of course be allowed for bigger values of $$C^{G_\mathrm{H}}_5$$. In particular for $$\delta =10^{-4}$$ the required value of the inverse radius is reduced by a factor $$10^{1/2}\simeq 3.2$$, and the value of $$\Lambda _S$$ is correspondingly reduced (considering that we are assuming $$d_T=2$$ largish dimensions with $$R_TM_s=3$$) by a factor of $$10^{5/6}\times (R_TM_s)^{-d_T/3}\simeq 2.6$$, with respect to the case of $$\delta =10^{-2}$$. This reduction on the value of $$\Lambda _S$$, with respect to the case of $$\delta =10^{-2}$$, can easily be compensated by increasing the value of $$\alpha _\mathrm{GUT}$$ and/or the value of the group factor $$C^{G_\mathrm{H}}_5$$, so that values as $$C^{G_\mathrm{H}}_5=4$$ might be preferred. The used values of 1 / *R* and the corresponding value of $$M_s$$ according to Eq. (3.11), for the case $$\alpha _\mathrm{GUT}\simeq \mathcal O(1)$$, are shown in the right panels of Figs. 5 and 6, from which we can see that $$M_s\gtrsim M_\mathrm{GUT}$$. The value of $$M_s$$ scales as $$\alpha _\mathrm{GUT}^{2/3}$$ so that for $$\alpha _\mathrm{GUT}=1/10$$ the value of $$M_s$$ should be reduced by a factor $$\sim 0.22$$, still in the ballpark of unification scales.

Finally, we would like to comment about the presence of the secluded $U(1)_\mathrm{H}$. At one-loop the messengers could a priori introduce a kinetic mixing between $U(1)_\mathrm{H}$ and the hypercharge $U(1)_Y$. This mixing vanishes in the simplest case of messengers in full representations of *SU*(5) and common mass as considered above. It is otherwise suppressed by the smallness of the hidden gauge coupling to be $\lesssim 10^{-5}$. A soft mass for $G_\mathrm{H}$ charged scalars living on the other end of the large dimension is induced at one-loop by the hidden gaugino mass as in [33, 34]. Their vacuum expectation value could then generate a mass for the $U(1)_\mathrm{H}$ gauge boson of order $\sim (\text {loop factor})^{1/2} g_\mathrm{H} /R \sim \mathcal O(10^7 \text { GeV)}$, where $g_\mathrm{H}$ is the hidden gauge group coupling. A quantitative discussion of the phenomenological and cosmological implications requires considering explicit models of the hidden sector, a subject which goes beyond the scope of this paper.

## $\mu /B_\mu$ terms

It is well known that gauge mediation cannot induce $\mu /B_\mu$ terms, as gauge interactions cannot generate them without direct couplings between the Higgs and messenger sectors [3]. However, gravitational interactions could do the job as in the Giudice–Masiero mechanism [16]. In this section we will illustrate this point by assuming that some particular effective operators are generated in the higher-dimensional theory.

The SS breaking can be understood in terms of supersymmetry breaking induced by the *F* term of the radion [35] superfield $\widetilde{T}$, with bosonic components.^10^

4.1 $$\begin{aligned} \widetilde{T}/M_s\equiv T=R M_s+\theta ^2 m_{3/2} ,\quad F_{\widetilde{T}}=m_{3/2}M_s \end{aligned}$$

where $m_{3/2}$ is the mass of the gravitino zero mode given in Eq. (2.1). The radion superfield then induces bulk gaugino $\lambda _\mathrm{H}$ masses through the coupling

4.2 $$\begin{aligned} \mathcal L=\frac{1}{4}\int \mathrm{d}^2\theta TW_{H\alpha } W^\alpha _\mathrm{H}. \end{aligned}$$

The radion superfield in Eq. (4.1) can also induce the $\mu$ and $B_\mu$ parameters required for electroweak breaking through effective operators as

4.3 $$\begin{aligned} \int \mathrm{d}^4\theta \{ [a^2\,|f(T)|^2+b\, f(T^\dagger )]H_1\cdot H_2+h.c.\} \end{aligned}$$

where $H_1$ and $H_2$ are the two Higgs doublets of the MSSM, *a* and *b* are real parameters and *f*(*T*) is a real function.^11^ Using (4.3) one can compute the values of the $\mu$ and $B_\mu$ parameters as

4.4 $$\begin{aligned} |\mu |&=\,m_{3/2}|f^\prime _{T}(\mathrm{RM}_s)||b+a^2 f(\mathrm{RM}_s)|\nonumber \\ B_\mu&=a^2\,m_{3/2}^2 |f^\prime _T(\mathrm{RM}_s)|^2 \end{aligned}$$

where we have to impose the phenomenological condition from electroweak symmetry breaking $|\mu |^2\simeq |B_\mu |$, which requires the relation

4.5 $$\begin{aligned} |b+a^2f(\mathrm{RM}_s)|\simeq |a|. \end{aligned}$$

For instance by using an asymptotic form (for large *T*) $f(T)\simeq T^{-n}$ and fixing $1/R=1/R(\omega )$ we can determine the value of the $\mu$ parameter for $a\simeq b\simeq 1$ as in the left panel plot of Fig. 7, where we plot contour lines of constant $|\mu |$ in GeV in the plane $(\omega ,n)$. The case $f(T)\simeq \log (T)$ is also plotted (right panel), for the case $a\simeq b\simeq \frac{1}{32\pi ^2}$. As we can see this case only works if both *a* and *b* parameters are loop suppressed so that condition (4.5) is also satisfied. In all cases the value of $M_s$ from the right panel of Fig. 5, which corresponds to $\alpha _\mathrm{GUT}=\mathcal O(1)$, has been used and the electroweak breaking condition $|\mu |^2\simeq B_\mu$ is fulfilled.

## Conclusion

In this work, we presented a novel mechanism of supersymmetry breaking where the SM gaugino, squark, and slepton masses arise predominantly from flavor blind gauge-mediated interactions, while the gravitino mass is superheavy due to an appropriate sequestering of the supersymmetry breaking sector. We have presented an example for how $\mu$ and $B_\mu$ parameters could be generated at the same time by effective supergravity interactions, as in the Giudice–Masiero mechanism.

Some important questions have been left aside. For instance, the radion stabilization was not discussed here. The potential generated for the modulus *T* should be such that at its minimum it reproduces the required hierarchy between the compactification and string scales. We are assuming here that the required stabilization mechanism will not perturb the main features of the mechanism presented here. Also, one needs to understand the origin of the effective operators describing the couplings of the radion to matter fields, as assumed in Sect. 4. We believe that such issues should be addressed in a more fundamental theory. The proposed mechanism should be in principle realized in string theory, since all basic ingredients we use already exist in type I constructions with intersecting D-branes, but all calculations have been done in the context of the effective field theory by summing over the contribution of the tower of KK modes. This can be tested by an explicit string implementation and model building which is left for future research as a very interesting open problem.

## Figures and Tables

**Fig. 8:**
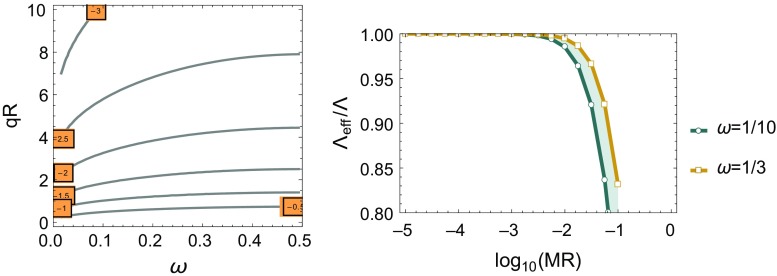
*Left panel* Contour lines of $\log _{10}[F(q)/F(0)]$. *Right panel* Plot of $\Lambda _\mathrm{eff}/\Lambda$ for different values of $\omega$

## Calculation of $\Pi (q^2)$

In Sect. 3 we have computed the seed of supersymmetry breaking for the calculation of gauge mediation as $F=\Pi (0)$ for zero external momentum. In this appendix we will compute $\Pi (q^2)$ for arbitrary external momentum *q*. To simplify the notation we will use inn this section units in which $1/R\equiv 1$ so all masses *m* are scaled as *mR*. We will also consider the case where $M\ll 1$ as in Sect. 3. The integral (3.3) is then generalized to

A.1 $$\begin{aligned} F(q)=\frac{\alpha _\mathrm{H}}{\pi }C_\mathrm{H}^\phi I(q) \end{aligned}$$

where

A.2 $$\begin{aligned} I(q)= & {} \int _0^\infty \mathrm{d}p^2 p^2 \sum _n\left[ \frac{M_n(\omega )}{p^2+M_n^2(\omega )}\frac{M}{(p+q)^2+M^2}\right. \nonumber \\&\left. -\frac{M_n(0)}{p^2+M_n^2(0)} \frac{M}{(p+q)^2+M^2}\right] \end{aligned}$$

and rotation to Euclidean momenta is performed. We now introduce integration over $\int _0^1\mathrm{d}x$ and the change of integration momentum $p\rightarrow p-q(1-x)$ and $p^2\rightarrow x\,p^2$

A.3 $$\begin{aligned} I(q)= & {} \int _0^1\mathrm{d}x\int _0^\infty \mathrm{d}p^2 p^2 \nonumber \\&\times \sum _n\left[ \frac{M_n(\omega )M}{\left[ M_n^2(\omega )+A\right] ^2}-\frac{M_n(0)M}{\left[ M_n^2(0)+A\right] ^2} \right] \end{aligned}$$

where

A.4 $$\begin{aligned} A=p^2+B,\quad B=q^2(1-x)+M^2(1-x)/x. \end{aligned}$$

After performing the summation over *n* and the integration over *p* one easily obtains

A.5 $$\begin{aligned} I(q)= & {} i\,\int _0^1 \mathrm{d}x \left\{ \sqrt{B}\log \left[ 1-e^{2\pi (\sqrt{B}-i\omega )}\right] \right. \nonumber \\&\left. +\frac{1}{2\pi } Li_2\left( e^{2\pi (\sqrt{B}-i\omega )}\right) +h.c.\right\} . \end{aligned}$$

In particular, for $M\ll 1$, $\sqrt{B}=q\sqrt{1-x}$, $I(0)=g_0(\omega )/4$, and we recover (as we should) the corresponding case studied in Sect. 3 (see Eq. (3.4)). Moreover, the function *I*(*q*) is dominated by its value at the $q=0$ region as we can see in the left panel of Fig. 8 where we plot contour lines of $\log _{10}[F(q)/F(0)]$ in the plane $(\omega ,q)$. We can see that, for any value of $\omega$, the function *F*(*q*) is dominated by its value at $q=0$.

In gauge mediation we define the supersymmetry breaking parameter $\Lambda \propto F/M$, which seeds the gaugino and scalar masses. We can compare $\Lambda$ with the value $\Lambda _\mathrm{eff}$ induced after integration of *F*(*q*) over the one-loop (for gaugino masses) or two-loop (for scalar masses) diagrams with internal momentum *q*. In view of the result of the left panel of Fig. 8 we expect that $\Lambda _\mathrm{eff}/\Lambda \simeq 1$ for $MR\ll 1$ as, in the integration over *q*, the momentum *q* is rescaled as $q\rightarrow qM$. This result is quantified in the right panel of Fig. 8 where we have computed the value of the parameter $\Lambda _\mathrm{eff}/\Lambda$ when $\Lambda _\mathrm{eff}$ is contributing to the one-loop gaugino masses. We can see that $\Lambda _\mathrm{eff}/\Lambda \simeq \mathcal O(1)$ is fulfilled for $MR\lesssim 0.1$. Then as we got from the right panel of Fig. 5 that $1/R\lesssim 10^{11}$ GeV, the previous condition translates into the mild condition $M\lesssim 10^{10}$ GeV, a region where the approximation done in Sect. 3 is fully justified.

## Footnotes

1 Here we are making the most conservative assumption of anarchic Kahler metric for the observable sector. This can be somewhat relaxed, and the strength of its corresponding gravitational suppression softened, in particular models, in the presence of a flavor symmetry. However, as the latter hypothesis is very model dependent, we will consider here the most conservative possibility of anarchic breaking of flavor symmetry by gravitational interactions.

2 We thank the referee for very useful comments leading us to consider new stronger constraints.

3
$\alpha _\mathrm{H}$ is the dimensionless four-dimensional coupling of gauge fields on a 4-brane wrapped along a cycle of length $\pi R$ in the extra dimension.

4 In fact the value of the parameter *F* actually depends on the value of the external momentum *q*, which we are taking here at $q=0$. The complete, more technical, analysis where the momentum-dependent supersymmetry breaking parameter *F*(*q*) is used to compute scalar and gaugino masses is left for Appendix A where we will demonstrate that the approximation of considering the insertion parameter *F*(0) is good provided that the mild condition $M\lesssim 0.1/R$ is fulfilled.

5 This amounts to assuming that all messengers in a given representation **r** of *SU*(5) are in the same representation of the hidden group $G_\mathrm{H}$ with quadratic Casimir operator $C_\mathbf{r }^{G_\mathrm{H}}$.

6 In particular, considering the small effect of the $\tau$ Yukawa coupling, the lightest slepton would be the $\tilde{\tau }_R$.

7 We are here imposing *R*-parity.

8 We can relax the allowed window by lifting the condition that $G_\mathrm{H}$ does not spoil the structure of *SU*(5) multiplets. This is a possibility we will not pursue in this paper.

9 These bounds could be evaded in non-minimal gauge mediation models, as in models of general gauge mediation [31].

10 We will use the dimensionless superfield *T*.

11 One can also introduce in (4.3) two different functions $f(T^\dagger )+|g(T)|^2$, but here we trade this freedom for the arbitrariness of the real coefficients *a* and *b*.
